# Brain Dynamics Underlying Preserved Cycling Ability in Patients With Parkinson’s Disease and Freezing of Gait

**DOI:** 10.3389/fpsyg.2022.847703

**Published:** 2022-06-16

**Authors:** Teja Licen, Martin Rakusa, Nicolaas I. Bohnen, Paolo Manganotti, Uros Marusic

**Affiliations:** ^1^Faculty of Medicine, Institute of Sports Medicine Maribor, University of Maribor, Maribor, Slovenia; ^2^Science and Research Centre Koper, Institute for Kinesiology Research, Koper, Slovenia; ^3^Division of Neurology, University Medical Centre Maribor, Maribor, Slovenia; ^4^Functional Neuroimaging, Cognitive and Mobility Laboratory, Department of Radiology, University of Michigan, Ann Arbor, MI, United States; ^5^Morris K. Udall Center of Excellence for Parkinson’s Disease Research, University of Michigan, Ann Arbor, MI, United States; ^6^Geriatric Research Education and Clinical Center, Veterans Affairs Ann Arbor Healthcare System, Ann Arbor, MI, United States; ^7^Department of Neurology, University of Michigan, Ann Arbor, MI, United States; ^8^Clinical Unit of Neurology, Department of Medicine, Surgery and Health Sciences, University Hospital and Health Services of Trieste, University of Trieste, Trieste, Italy; ^9^Department of Health Sciences, Alma Mater Europaea—ECM, Maribor, Slovenia

**Keywords:** freezing of gait (FOG), Parkinson, cycling, cortical oscillations, beta band

## Abstract

Parkinson’s disease (PD) is generally associated with abnormally increased beta band oscillations in the cortico-basal ganglia loop during walking. PD patients with freezing of gait (FOG) exhibit a more distinct, prolonged narrow band of beta oscillations that are locked to the initiation of movement at ∼18 Hz. Upon initiation of cycling movements, this oscillation has been reported to be weaker and rather brief in duration. Due to the suppression of the overall beta band power during cycling and its continuous nature of the movement, cycling is considered to be less demanding for cortical networks compared to walking, including reduced need for sensorimotor processing, and thus unimpaired continuous cycling motion. Furthermore, cycling has been considered one of the most efficient non-pharmacological therapies with an influence on the subthalamic nucleus (STN) beta rhythms implicative of the deep brain stimulation effects. In the current review, we provide an overview of the currently available studies and discuss the underlying mechanism of preserved cycling ability in relation to the FOG in PD patients. The mechanisms are presented in detail using a graphical scheme comparing cortical oscillations during walking and cycling in PD.

## Introduction

Parkinson’s disease (PD) is considered one of the most common degenerative diseases of the nervous system that affects movement (Snijders et al., 2011; Evens and Clark, 2017). The primary manifestations of PD are tremors, slowness of movement (bradykinesia), stiffness of the muscles, postural instability, gait difficulties, and lack of facial expressions (Müller et al., 2019). Bradykinesia, in particular, is considered a major cause of motor difficulties, affecting almost all activities of daily living (Hallett, 2011). Since there is no cure for PD, current pharmacological therapies can only mitigate symptoms but do not have disease-modifying effects. Moreover, as PD progresses, the therapeutic window for medical therapies narrows with an increased risk of side effects (Shah et al., 2016).

About a decade ago, Snijders et al. (2012) made the novel discovery of cycling ability being preserved not only in PD patients without freezing of gait (FOG) but, interestingly, also in those grounded by severe freezing gait disturbances (Hallett, 2011; Snijders et al., 2011, 2012). Besides the activation of the classical motor control regions (i.e., motor, somatosensory, premotor, and supplementary motor cortex, as well as cerebellum), cycling at various intensities in healthy subjects has shown to activate regions associated with autonomic regulation as well (e.g., insular cortex) (Bodnariuc Fontes et al., 2015; Fontes et al., 2020). Furthermore, the anti kinetic nature of the oscillatory beta activity has been discussed as a possible factor to gait impairments in PD patients (Storzer et al., 2017) and a possible explanation of why the cycling ability is better preserved than walking in PD patients, especially those with FOG. Although walking and cycling appear similar due to similar lower extremity movements involving the same muscle groups, these two movements have been found to be associated with functional differences in the activation of motor networks of cerebral structures, including the basal ganglia (Storzer et al., 2016).

As the availability of new brain imaging techniques allows the assessment of brain dynamics in real-time settings and the number of studies investigating the underlying cortical oscillations during cycling and walking with PD patients is increasing, it is important that this information is summarized to provide an overview of the literature. The aim of this review is therefore to highlight the differences in cortical oscillations between walking and cycling, more so to summarize the literature that has examined the underlying electrophysiological mechanisms of preserved cycling ability compared to FOG in PD patients.

## Freezing of Gait

Freezing of gait is defined as the episodic inability to move forward despite the intention to walk (Nutt et al., 2011). This broad unique and disabling clinical phenomenon has been identified as one of the main contributors to gait disturbances, resulting in a substantial reduction in step length at a higher cadence (Chee et al., 2009) and more frequent leg tremors during FOG episodes (Nutt et al., 2011).

Theoretical considerations suggest that the freezing phenomenon is associated with cortical dysfunction across multiple levels of the locomotor and postural control systems, including cortical, subcortical, and brainstem regions (Fox et al., 2018; Müller et al., 2019). It is a known fact, that the oscillatory activity in the cortical network (Rakusa et al., 2018) and cortico-basal ganglia loop, particularly in the beta band range (13–35 Hz), promotes maintenance of motor state at the expense of voluntary movement, where a pathological synchronization of beta frequency in the basal ganglia of PD patients has been observed (Oswal et al., 2016; Fox et al., 2018). This exaggerated beta-band activity is linked to slowed movements and therefore, associated with bradykinesia (Wichmann, 2019). Likewise, single STN neurons have been most strongly synchronized to beta oscillations in frontal and midline cortical areas with stable time delays that persist during periods of self-paced movements. Furthermore, the size and intensity of the STN phase locking to beta oscillations recorded over the mesial cortex have been highly correlated with the severity of akinetic motor symptoms, which all together suggests, that persistent synchronization of STN neurons to premotor cortical beta oscillations is a candidate mechanism for akinetic symptoms in PD (Sharott et al., 2018). Interestingly, the gamma band amplitude has been observed to be uncommonly connected to the activity in the beta band and thus associated with akinesia as well (Wichmann, 2019). While non-freezers with PD exhibited a reduction in power across the beta band in STN during walking, freezers showed an increased oscillatory activity specifically in the low beta band (12–22 Hz) (Singh et al., 2013). It has been suggested, that the ∼18 Hz oscillation frequency may reflect a movement-inhibitory signal throughout the motor network, as it has been observed to dominate in the dopaminergic medication “off” state and is correspondingly reduced by both levodopa medication and deep brain stimulation (Oswal et al., 2016). Therefore, it may represent a key signature of altered basal ganglia activity, which differentiates PD patients with FOG from those without FOG irrespective of the motor state (López-Azcárate et al., 2010), resulting in reduced movement automaticity and FOG episodes.

Furthermore, the STN receives direct cortical projections from frontal areas through the hyper-direct pathway (Wang et al., 2020), which is assumed to mediate a top-down signal that may block movements (Aron et al., 2016). Withal, the increased cortical activity of the alpha band (7–11 Hz) has been observed during upper limb FOG episodes in PD patients, which has been recognized as the possible explanation of the increased cortical motor inhibition; thus the transition from idle rhythm to the actual movement (Scholten et al., 2016). The increased cortical activity of the alpha band indexes the inhibition of incoming somatosensory information (Klimesch, 2012). The alpha rhythm has been found not to be specific to the visual system but to reflect a general mechanism for resource allocation in the brain that reflects a top-down attentional bias with a profound impact on performance in the sensorimotor system (Jensen et al., 2019).

Correspondingly, as dopaminergic denervation in the basal ganglia is responsible for a reduction in movement automaticity in PD patients, their reliance on attention to executing such movements increases (Wu and Hallett, 2005; Lewis and Shine, 2016). This results in a slower or lower amplitude execution of movements required for almost all everyday behaviors (Wu and Hallett, 2008). Therefore, the tasks that redirect the PD individual’s attention from the walking itself (i.e., dual-task (DT) paradigms) have been shown to commonly exacerbate freezing episodes in PD patients, due to the added requirement of more attentional attention, sensory-motor information processing through an altered cortical-basal ganglia loop (Lang and Bastian, 2002), whereas, focusing on stepping will alleviate it (Nutt et al., 2011). In the same way, FOG commonly occurs when PD patients walk through narrow doorways, where the step length decreases, with the phenomenon worsening as the size of the doorway decreases (Snijders et al., 2012). The effect sizes are variable and strongly rely on the nature of the task, cohort selection and medication state (Vervoort et al., 2016). Therefore, exaggerated loss of automaticity and/or cognitive deficits may result in decreased ability to divide attention while dual-tasking, resulting in an altered, less automatic walking pattern or freezing motor behaviors (Abe et al., 2003).

## Pathophysiological Mechanism of Cycling

For the motor cortex to enable an ongoing motor process, it relies on the somatosensory input from the peripheral sensors like joint receptors, muscle spindles and cutaneous reflexes. As the proprioceptive system has been found to be impaired in PD patients, this results in a lack of peripheral afferent input and disturbed motor output (Conte et al., 2013). It is hypothesized that a lower amount of intrinsic information processed by the brain during cycling may result in less disruption of cortical basal ganglia circuitry, reducing the pathological beta-band synchronization observed in PD patients (Snijders et al., 2011, 2012; Storzer et al., 2017). Intrinsic information in this case includes information from balance sensors and postural control in conjunction with to muscle and tendon control. Therefore, one of the explanations for preserved cycling ability can be found in the continuous nature of cycling and the general absence of continuous stationary phases during walking (Snijders et al., 2011; Storzer et al., 2017). The somewhat restricted movement patterns of cycling, in which the pedals move in a fixed, symmetrical range of motion, may simplify leg coordination compared to walking and require less motor planning and a smaller in the motor areas of the pedal cycle (Storzer et al., 2017).

Therefore, a lower amount of information processed by the brain during cycling could reduce the risk of obstructed signal between the cortico-basal ganglia loops and thus reduce the risk of FOG (Lewis and Barker, 2009). In the same way, FOG was found to be partially alleviated by using a “walk-bicycle,” also known as a bicycle without pedals and with a low seat (Stummer et al., 2015). As even though the locomotion on the walk-bicycle requires alternating stepping movements of the legs, it seems to reduce the need for postural control and lateral weight shifts that were shown to be abnormally coupled in patients with FOG (Adkin et al., 2003; Jacobs et al., 2009). However, when the movement sequence of cycling is made more complicated, such as removing the pedals from the bike, this can increase cognitive load, which can lead to irregular cycling patterns and the return of FOG episodes in PD individuals (Abe et al., 2003; Ridgel and Ault, 2019). Although bilateral coordination of limb movements has been highlighted as a susceptible factor to cognitive load (Keus et al., 2007; Snijders et al., 2012), the reduced amount of information processed by the brain in the motor networks is thought to be the most likely reason why cycling abilities are not impaired in most PD patients, including those with FOG (Snijders et al., 2011, 2012).

External tactile cues (e.g., auditory or visual), triggered by the appropriate rhythmic cycling movements of the pedals, are thought to play an important role in the preservation of cycling ability in PD patients (Snijders et al., 2012). The sensory feedback produced by the symmetrically moving pedals can also regulate the amplitude and timing of the leg movements during cycling (Snijders et al., 2011). Tactile cues have also been shown to be an effective tool to overcome the defective basal ganglia circuitry of PD patients, by activating the motor cortex *via* alternative circuitries (Keus et al., 2007; Nieuwboer, 2008; McKee et al., 2020).

## Cortical Oscillations During Walking and Cycling in Parkinson’s Disease Patients

Despite the fact, that there are some similarities in cognitive functions, muscle groups activated and pace, walking and cycling differ in sensorimotor input, coordination, postural control and involve different cerebral structures in the motor networks of cerebral structures, particularly the basal ganglia (Storzer et al., 2016). It should be noted that the exact complex physiology behind the preserved ability to cycle in patients with PD, especially in patients with FOG, remains poorly understood. PD patients with FOG have been identified with a selective, prolonged, and narrow band beta oscillation locked to the initiation of movement at ∼18 Hz. This oscillation was observed irrespective of the actual FOG episode, as well as being descriptively weaker and rather short in duration upon initiation of cycling, suggesting that it most likely reflects susceptibility to freezing. Similarly, walking requires a well-regulated unity between motor commands and anticipatory postural adjustments, including alternative weight shifts between the legs, which are impaired in patients with FOG (Vandenbossche et al., 2013; Sacheli et al., 2018). In addition, balance deficits also in the backward direction, which is essential for human upright gait (Carpenter et al., 2004), have been found to be impaired in PD patients, implying that cycling may represent a type of exercise under “facilitated conditions” from the perspective of PD patients (Snijders et al., 2011; Ridgel and Ault, 2019). This suggests that walking and cycling access different cerebral structures or pathways. To understand this rare abnormality, it is essential to investigate specific correlations and differences in cortical oscillations during walking and cycling (Seeber et al., 2014; Sacheli et al., 2019).

Walking and cycling have been associated with a decrease of beta power over the motor cortex and SMA locked to movement initiation (Seeber et al., 2014; Storzer et al., 2016). Correspondingly, Storzer et al. (2017) found specific oscillatory dynamics in the low beta band range in local field potentials recordings from the STN in PD patients with FOG, but not in those without FOG when comparing walking and cycling. This was the first time a narrow band oscillatory signal was demonstrated in direct relationship to FOG. However, PD patients showed an equally strong beta rebound upon movement termination after walking and cycling, suggesting that the difference in beta power suppression between cycling and walking is more specific for the duration of movement (Storzer et al., 2017). Therefore, as beta power decrease seems to be somehow related to elevated cortical excitability, a stronger beta-band oscillatory decrease during the execution of the cycling movement, as well as a faster beta rebound after movement termination compared to walking, has been proposed as one of the main features of the pathophysiological mechanisms that explain why the ability to ride a bike is still preserved in patients with FOG, as well as an important role for the functional difference of the types of movement (Storzer et al., 2017).

**FIGURE 1:**
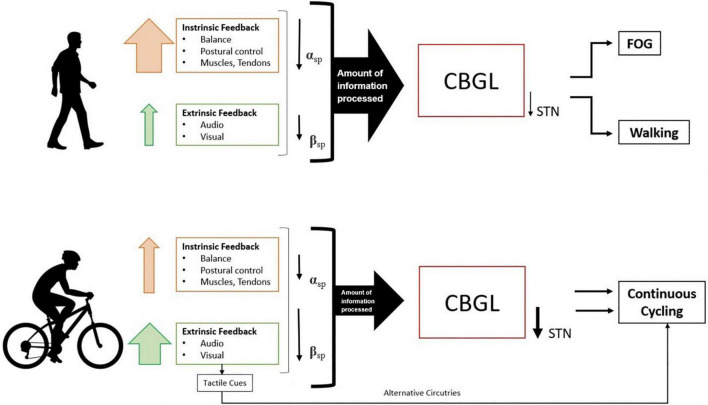
Differences in information processing and brain oscillations during initiation and continuous movement in walking and cycling. α_sp_ = alpha-band suppression; β_sp_ = beta band suppression, CBGL, cortico-basal ganglia loop; STN, subthalamic nucleus; FOG, freezing of gait. The size of the arrow presents the degree of effect.

Furthermore, walking has been correlated with greater suppression of alpha power (Storzer et al., 2017), which has been generally associated with somatosensory processing (i.e., attention and multi-sensory information processing) (Klimesch, 2012). This suggests that alpha suppression during walking is closely related to the increased amount of intrinsic and extrinsic sensory feedback (Wagner et al., 2014), as walking requires more processing of extrinsic information to regulate the appropriate gait cycle throughout the movement execution. Accordingly, walking compared to cycling is thought to have increased sensory and attentional demands due to greater muscle recruitment and sensorimotor integration of important sensory feedback throughout movement execution (Wagner et al., 2014). On the other hand, cycling is thought to place fewer demands on postural control and coordination functions, presumably resulting in a smaller amount of information processed by the brain and thus less strain on the striato-thalamo-cortical loop and greater suppression of the beta band during cycling than during walking (Storzer et al., 2017). Cycling may have an additional advantage where external tactile cues from the pedals may help overcome the defective cognitive basal-ganglia loop of PD patients by activating the motor cortex through alternative pathways, thus switching automatic motor programs to externally controlled processes (Snijders et al., 2011, 2012; Figure 1).

Functional differences in the cerebral networks underlying cycling and walking would be expected as these two physical activities differ in some sensorimotor input, postural control and coordination functions (Alberts et al., 2011; Storzer et al., 2017). Postural control during standing is impaired in PD patients, with more significant deterioration in PD patients with FOG (Lewis and Barker, 2009). As PD patients, particularly those with FOG, have difficulty generating preparatory lateral weight shifts, such anticipatory adjustments appear to be less critical during cycling due to the rhythmic pedal movements (Jacobs et al., 2009). Dynamic balance control, which is often altered in PD patients, may represent an additional important aspect in overall movement generation and execution, given the differences between cycling and walking. While walking mainly requires dynamic balance control in the forward-backwards direction, cycling on the other hand, mostly requires lateral weight shifts from one pedal to the other (Zehr et al., 2007). Several studies have shown that balance deficits in PD patients are direction-dependent, being greatest in the forward-backwards direction and less pronounced in the lateral direction (Carpenter et al., 2004). Therefore, the relatively preserved mediolateral stability and less demand for front to back stabilization during cycling vs. walking may present a key reason for PD patients’ preserved ability to cycle (Snijders et al., 2011, 2012; Shah et al., 2016).

### Limitations

Potential limitations of this review article include the limited number of studies that examined cortical changes in PD patients while cycling through various interventions and even fewer studies that compared cortical changes between walking and cycling. Although the studies used different methods to track and measure cortical oscillations, it is still possible to compare the results. However, the results were mostly based on a small number of subjects, making it difficult to generalize the results to the larger PD population. In addition, there was a lack of direct comparison of PD patients with FOG with those without FOG, as some of the studies compared PD patients with healthy subjects independent of FOG. Therefore, future studies are needed to investigate the complex dynamics in PD patients during locomotion and cycling in more ecologically valid settings.

## Conclusion and Implications

Overall, cycling ability remains unaffected in most PD patients with or without FOG and has been characterized by a stronger movement-related suppression of pathological beta oscillations compared with walking, with an influence on STN beta rhythms attributable to the effects of deep brain stimulation. It has been suggested that a stronger wide band beta suppression during cycling reduces the abnormal ∼18 Hz oscillation in the STN in PD patients with FOG and allows continuous movement during cycling. This is because the movement is continuous and the brain processes less information in the motor networks compared to walking. Cycling is not only easier for PD patients, but also improves PD motor symptoms.

## Author Contributions

TL contributed to the conceptual idea, reviewed and analyzed the literature, and wrote the manuscript. UM provided the conceptual idea and reviewed and edited the manuscript. MR, NB, and PM were responsible for editing the manuscript and critical evaluation. All authors approved the final version of the manuscript.

## Conflict of Interest

The authors declare that the research was conducted in the absence of any commercial or financial relationships that could be construed as a potential conflict of interest.

## Publisher’s Note

All claims expressed in this article are solely those of the authors and do not necessarily represent those of their affiliated organizations, or those of the publisher, the editors and the reviewers. Any product that may be evaluated in this article, or claim that may be made by its manufacturer, is not guaranteed or endorsed by the publisher.
